# The green side of splicing: algal spliceosome shows remarkable structural conservation

**DOI:** 10.1038/s44318-025-00403-6

**Published:** 2025-03-06

**Authors:** Wojciech P Galej

**Affiliations:** EMBL Grenoble, 71 Avenue des Martyrs, 38042 Grenoble, France

## Abstract

A new study solves the Cryo-EM structure of a *Chlamydomonas reinhardtii* spliceosome.

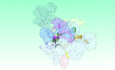

## Teaser

Spliceosome structures have been investigated extensively in the past decade, but most of the studies have been restricted to a handful of model organisms. New research by Lu et al, looks outside this field-of-view, reporting on the structure of the spliceosome from a unicellular green alga, *Chlamydomonas reinhardtii*, showing its extraordinary structural conservation.

The spliceosome is one of the cell’s most complex molecular machines, which catalyses the removal of non-coding introns from precursors of messenger RNAs in a process known as pre-mRNA splicing. Several decades of research have laid the foundations for mechanistic studies of pre-mRNA splicing. However, only when cryoEM structures of the spliceosome became available, the architectural and mechanistic insights into pre-mRNA splicing started to emerge (Kastner et al, 2019; Tholen and Galej, 2022). Despite rapid development of the entire field, structural studies of the spliceosome are mostly restricted to a few model organisms, such as *Saccharomyces cerevisiae, Schizosaccharomyces pombe* and *Homo sapiens*, and a handful of model pre-mRNA substrates. Using different species can facilitate trapping transient intermediate states, as recently demonstrated by the study of the post-splicing Intron-Lariat Spliceosome complex from *Caenorhabditis elegans* (Vorländer et al, 2024).

**Figure 1:**
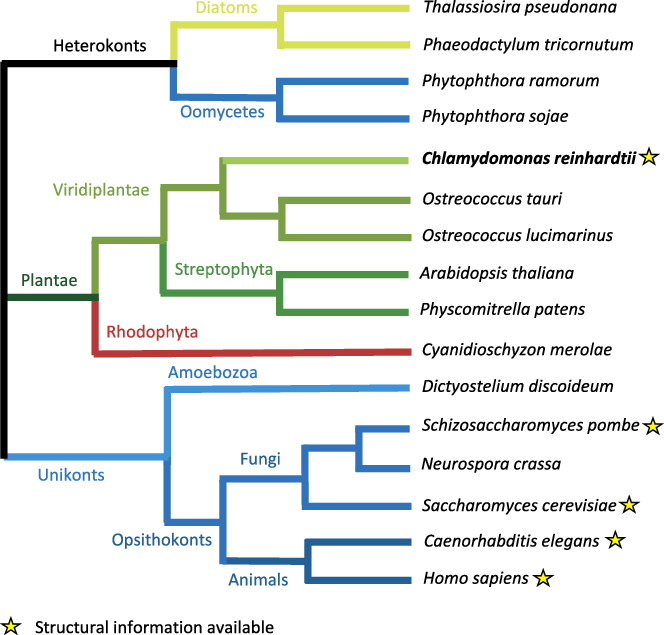
Evolutionary tree of life highlighting (stars) model organisms for which at least one spliceosome structure has been determined experimentally. Adapted and modified with permission from Merchant et al (2007).

In the current issue of *EMBO Journal*, Lu et al (2024), investigate the spliceosome structure in *Chlamydomonas reinhardtii*, a unicellular green algae. Diverging from land plants over a billion years ago (Fig. 1), *Chlamydomonas* has a unique position in evolution as it contains many genes that are specific to both plant and animal lineages (Merchant et al, 2007). *C. reinhardtii* has an intron-rich genome, whose organisation and exon–intron structure resemble that of humans and land plants (Table 1). Exons of *C. reinhardtii* undergo alternative splicing, although to a much lower extent than in land plants. This makes it an interesting model organism to study alternative splicing regulation in a simplified system.

**Table 1:** Exon–intron architecture of the genomes of selected model organisms.

Organism	Genes with introns [%]	Avg. Exons per gene	Avg. Intron length	Avg. Exon length
*Chlamydomonas reinhardtii*	88	7.4	336	240
*Saccharomyces cerevisiae*	5	1	256	1500
*Schizosaccharomyces pombe*	43	2	107	330
*Arabidopsis thaliana*	79	5	165	304
*Homo sapiens*	85	9	3400	282

Reproduced from Labadorf et al (2010).

In the current study, Lu et al, purified native splicing complexes from *C. reinhardtii* using affinity tags on the components of the NTC/NTR complex (present in the spliceosome from its activation to disassembly) and analysed them by single-particle cryoEM. Structural analysis revealed that the purified complex is in the C* configuration; that is, it is ready for exon ligation, but the 3’-exon of the pre-mRNA is not yet engaged at the active site. The visualised core of the *C. reinhardtii* spliceosome is nearly identical to the core of the yeast and human C* complexes (Fica et al, 2017; Bertram et al, 2017), showing extraordinary structural conservation between the RNA catalytic cores and numerous scaffolding proteins.

The authors of the study discuss several subtle “metazoan-like” features of the *C. reinhardtii* spliceosome, including the presence of U5-40K protein, Aquarius helicase (Schmitzová et al, 2023), and an extended 16 nt-long duplex between the U6 snRNA and 5' splice site, all of which have been seen in the structures of human C* complexes. Another noteworthy observation is that the splicing factor, Prp17, is relatively well-resolved in *C. reinhardtii*, when compared to reports from other species. This could be potentially due to stabilising interactions between its N-terminal helix and a relatively short 5’ stem-loop of the U6 snRNA, which are unique to *C. reinhardtii*.

The conserved exon ligation factors (i.e. Prp18, Slu7 and Prp22) are missing in the reported structure, even though they are encoded by the *C. reinhardtii* genome. This could be caused by a relatively weak binding affinity in this organism or a result of the heterogeneity arising from undefined pre-mRNA species associated with this natively purified sample. If the former is true, it could explain why the authors managed to capture and enrich this particular complex, which has not undergone a full splicing cycle and subsequent disassembly. A thorough proteomic analysis could help address this question in the future. It is also possible that the purified complex is bound to a subset of inefficient and/or slow-splicing pre-mRNA substrates; here, analysis of its RNA composition could provide some crucial insights.

Recent reports of the human C* complexes revealed that numerous additional protein factors (such as Cactin, FAM32A and PRKRIP1) can regulate exon ligation (Zhan et al, 2022; Dybkov et al, 2023). There is no evidence of the presence of any of these additional C* factors in the structure resolved by Lu et al, and whether any have functional homologues in the *C. reinhardtii* remains to be seen.

Taken together, the work by Lu et al, provides a first into the core of the plant splicing machinery, revealing its remarkable structural conservation and extremely close similarity to evolutionarily distant organisms. At the same time, many questions related to splicing biology in *Chlamydomonas*, and more broadly, other plants, remain unanswered. For example, are there any plant-specific features and adaptations of the spliceosome? How do they affect its function, and can such features be exploited to advance our mechanistic understanding of pre-mRNA splicing? This and other questions remain to be investigated in the future.
